# Whole Grains and Phenolic Acids: A Review on Bioactivity, Functionality, Health Benefits and Bioavailability

**DOI:** 10.3390/nu10111615

**Published:** 2018-11-01

**Authors:** Lavinia Florina Călinoiu, Dan Cristian Vodnar

**Affiliations:** Faculty of Food Science and Technology, Institute of Life Sciences, University of Agricultural Sciences and Veterinary Medicine of Cluj-Napoca, Calea Mănăştur 3-5, 400372 Cluj-Napoca, Romania; lavinia.calinoiu@usamvcluj.ro

**Keywords:** whole grains, bran, phenolic acids

## Abstract

Cereal grains represent one of the major sources of human food and nowadays, their production has increased to fulfill the needs of the world’s population. Among whole grains, wheat is the most popular and contributes significantly to the human diet. Whole grains possess great nutritional and bioactive properties due to their fractions, bran and germ, that comprise unique health-promoting bioactive components. The evidence of health benefits in human intervention studies, as well as a World Health Organization report for 2012–2016, supports the dietary consumption of whole grains and whole-grain foods. The inverse correlation between whole grain consumption and the reduced risk of chronic diseases and metabolic syndromes was underlined by several epidemiological studies. This article focuses on the bioactive components of whole grains and their fractions, namely phenolic acids, starting from their chemical structure, bioactivity and bioavailability. According to the conclusive evaluation of the human intervention studies conducted using cereal bran and whole grains intake, the assumption that the bioactive compounds determine health outcomes is illustrated. In the last part of the work, the functional potential and the health claims related to whole grains and bran intake are discussed, as well as new technologies and strategies to enhance their health potential by an increased bioavailability.

## 1. Introduction

According to Eurostat (2016) [1], cereal grains represent one of the world’s major sources of food contributing up to 300 million tons annually. In particular, wheat is the major cereal produced and its grain products are highly consumed worldwide [2]. Whole grains make up a significant group, which possesses great nutritional and bioactive properties due to its fractions, bran and germ, that comprise unique health-promoting bioactive components [3,4,5], presenting a more complex and beneficial nutritional profile than refined grains [6]. These considerations have increased researchers’ interest in exploring whole grain effects on human health and their bioactive fractions as potential sources for functional food and ingredients. Based on the World Health Organization report for 2012–2016 [7], consumption of whole grains may decrease the risk of non-communicable diseases (e.g., type 2 diabetes, cardiovascular disease, hypertension and obesity), therefore a number of epidemiological studies, precisely large prospective studies that now include millions of person over years of follow-up, have underlined the inverse correlation between whole grain consumption, including the bran part, and the reduced risk of chronic diseases and metabolic syndromes [8,9,10,11,12,13,14,15]. In addition, it was constantly reported that whole-grain foods have relevant biological activity in humans [16] with bran and aleurone as significant bioactive fractions [17], except when they are perceived as byproducts of the flour milling industry; therefore, being discarded and destined for animal feeding [18]. Instead, they comprise most of the micronutrients, fiber, and phytochemicals of the grain that could significantly impact on the nutritional quality of human food if integrated in flours or used as food ingredients [19]. Emerging research suggests that phenolic acids may, together with fiber, be responsible for many of the health effects of whole grains [20,21,22,23] and their derivatives account for about one-third of the total intake of polyphenols in our diet [24]. Consumption of 2–3 servings per day (~48 g) of whole grains may reduce the risk of cardiovascular disease, cancer, and type 2 diabetes mellitus [25,26]. Scientific studies revealed that bran and germ fractions exhibit their positive health effects on both animal and humans by two mechanisms: First by releasing the indigestible fibers to modulate gut microbiota composition and activities; and second by delivering substrates, such as resistant starch, nonstarch polysaccharides (β-glucan and arabinoxylans) and phenols to be metabolized into practical microbiota metabolites [6].

The cereal bran is a major source of phenolic acids-antioxidants, fibers and minerals, whereas aleurone is the critical component generally overlooked in favor of indigestible fiber. Otherwise, it comprises the highest amount of bioactive compounds exhibiting significant antioxidant activity [18] with ferulic acid as its major antioxidant [10]. In cereals bran, besides being a cheap and readily available by-product of the cereal industry, its concentrated source of phenolic compounds have anti-inflammatory properties that can act beneficially on the gastrointestinal tract. The intake of whole grains may lower the incidence of colon cancer [27]. Particularly, wheat bran is rich in phenolic acids, which are mainly covalently cross-linked with cell wall polymers [28]. In order to exhibit their health-related positive impact, phenolic acids have to resist food-processing conditions, be released from the food matrix, and be bioaccessible in the gastrointestinal tract, subject to metabolism, and reach the target. Therefore, the current trend on bioavailability and bioaccessibility, as well as valorization of waste compounds is becoming more and more popular [29,30]. Bioavailability, from its nutritional side, refers to the efficient use of nutrients and bioactive compounds by the body, while bioaccessibility involves the released solubilized fraction into the gastrointestinal fluid that has become available for intestinal uptake [31]. Bioavailability is assessed by in vivo studies of blood and/or urine metabolites after consumption of targeted compounds, while bioaccessibility is determined by in vitro studies that analyze the amount of compounds available for intestinal uptake [32]. Researchers are searching for strategies and processing technologies to enhance the content and bioavailability of nutrients and bioactive compounds of cereal foods. The bioavailability of compounds depends on bioaccessibility, absorption, transformation, disposition and excretion, where the main issue is bioaccessibility, which is affected by how food processing influences nutrients available for digestion and absorption in the GI tract [24]. In order to validate phenolic acids bioactive potential in humans and their dietary importance in already processed food, an assessment of their changes during processing is also necessary [6]. Based on the recent findings on the health-related effects of bran components, the approach of using it as a functional food ingredient in bakery and pasta processes is of major interest [33,34], as well are the strategies to increase their phenolic acids bioaccessibility/bioavailability.

This manuscript seeks to review the recent scientific literature concerning whole grains and their fractions, bioactive compounds and nutrients, with a focus on bran phenolic acids. Their biological activities, health outcomes, health claims, functional potential and bioavailability/bioaccessibility implications will be discussed. This review focuses on works assessing bioaccessibility and bioavailability, food processing influence and recent strategies and technologies to unlock phenolic compounds bioaccessibility and bioavailability. Finally, this work is intended to encourage new research in an area with promising findings in the near future.

## 2. Whole Grains and Their Main Fractions

Wheat, rye, rice, oats or barley are among the major whole grains representing a major source of food for humans since old times. All these grains are structurally similar and divided into three distinct fractions: The outer fiber-rich bran, the micronutrient-rich germ and the starchy main ‘body’ known as the endosperm [35]. After food processing, the kernel has to keep the same ratio of bran, germ and endosperm as the original grain in order to be declared as “whole grain” [36]. In 2006, the United States (US) Food and Drug Administration (FDA) approved the whole grain label, but currently each country or responsible association/organization is updating their definition for whole grain and whole-wheat products, as they are different [2]. There is a strong need for a general approved definition of whole grain and whole-grain foods, as well as knowledge on their health related-compounds in food and their positive impact after intake [17].

**Figure 1:**
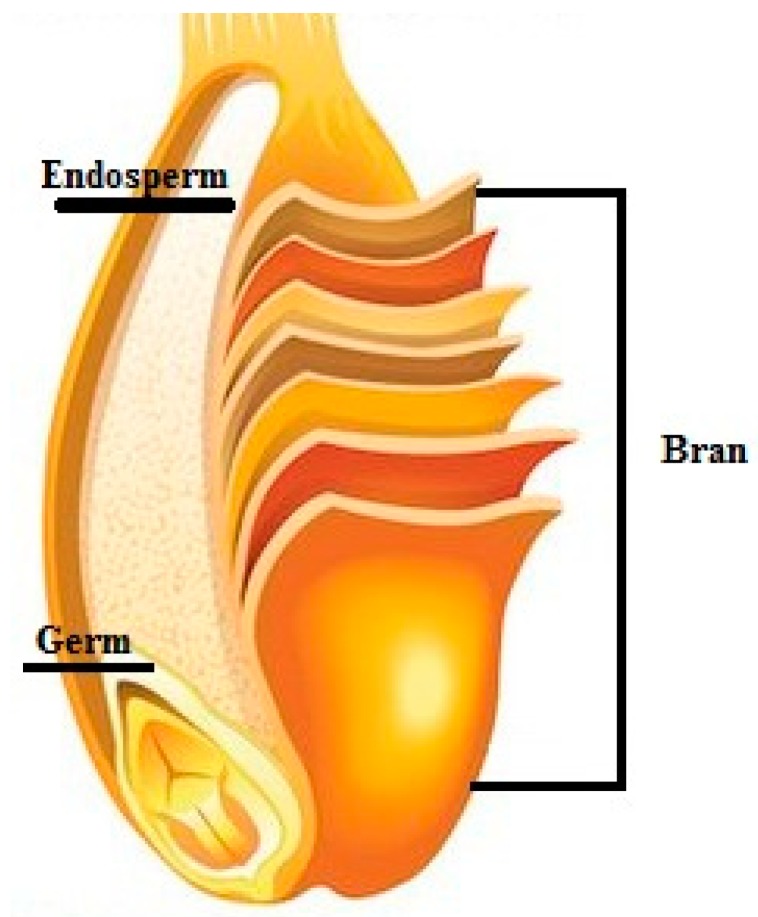
Whole-wheat grain main sections (adapted from Reference [37]).

In the Figure 1 below, the main sections of whole grains are illustrated.

The bran is the outer skin composed of multiple layers, which contains fiber, minerals, vitamins and bioactive compounds, among which phenolic acids are of interest being classified as bioactive phytochemicals that have important health effects on humans [38]. The germ is the embryo, which contains essential fatty acids, B vitamins, vitamin E, selenium, and antioxidants. The endosperm, having the largest size of the kernel, comprises mostly starchy carbohydrates, like glucose [17].

The amount of nutrients and bioactive compounds is strongly influenced by grains species, the cultivar used, and the growing conditions [17]. The nutritional profile of several grain species and their whole grain and refined version are illustrated in Table 1 below.

### Bioactive Compounds

The significant bioactive compounds in whole-grain cereals are phenolic compounds among which ferulic acid and cinnamic acid are representative; dietary fibers, like beta-glucan; lignans, phytic acid, inositols and betaine [36,41,42].

**Figure 2:**
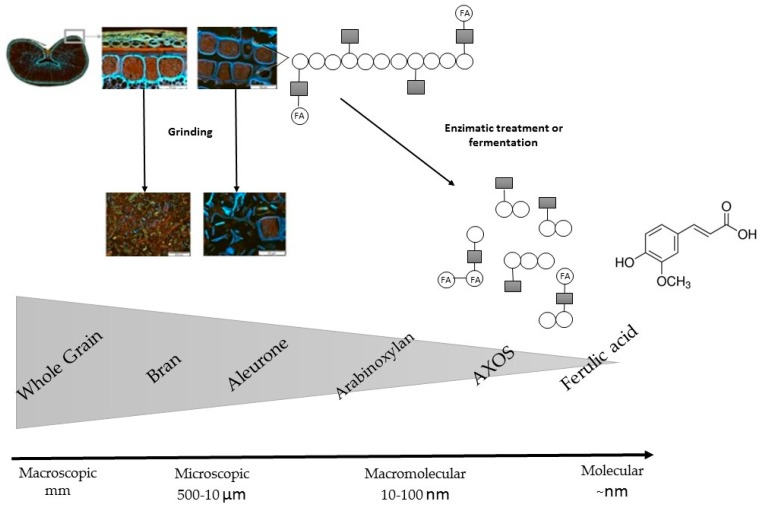
From macro to molecular levels, the most nutritionally interesting technological fractions of wheat bran and aleurone layer, as well as arabinoxylan and ferulic acid components (adapted from Reference [43]).

The structures of the whole-wheat grain, bran and aleurone layer, and their main dietary fibers—arabinoxylan together with arabinoxylan oligosaccharides (AXOS), and phenolic acid—ferulic acid components were illustrated by Sibakov et al. (2015) [43] in the following Figure 2. The food processing effects (grinding, enzymatic hydrolysis or microbial fermentation) on their structures are also presented.

The phenolic compounds are found under glycosides forms linked to different sugar moieties, or as other forms linked to organic acids, amines, lipids, carbohydrates and other phenols, whereas the major ones are phenolic acids.

## 3. Phenolic Acids

Phenolic acids are among the majority metabolites of cereal crops with antioxidant effects on humans [44,45] and they are of a major importance as they represent 30% of total phenolic acids in Mediterranean diets [18].

Phenolic acids are a specific class of polyphenols which are usually involved in mechanisms of defense against biotic and abiotic stresses [46]. The phenylalanine serves as the substrate for the initiation of phenolic acids biosynthesis via the phenyl propanoid pathway [47]. The biotic and abiotic factors, like environmental issues and agronomic strategies, can influence the biosynthetic pathway, therefore the phenolic acid content may vary [48]. Additionally, the genetic influence must be considered, as the genetic-environmental interactions may result in a large phenolic content fluctuation among cereal species and cultivars of the same species [49,50].

The phenolic acids occur in free, conjugated or insoluble bound forms [51], whereas about 95% of grain phenolic compounds (PC) are ester- or ether-linked to cell wall polysaccharides and cross-link them intra- and/or intermolecular to form networks. They are also indicated as dietary fiber-phenolic compounds (DF-PC) [52]. The whole grains phenolic acids are divided into hydroxybenzoic acids and hydroxycinnamic acids, based on C1–C6 and C3–C6 skeletons, respectively [44]. The hydroxybenzoic acid derivatives comprise *p*-hydroxybenzoic, vannilic, syringic, and gallic acids while the hydroxycinnamic acids include ρ-coumaric, caffeic, ferulic, and sinapic and occur in the form of esters and glycosides. Ferulic acid is the major hydroxycinnamic acid. The other derivates, as well asbenzoic acid derivatives, occur in smaller amounts. The structures of cinnamic and benzoic acids derivatives are presented in the Table 2 and Table 3 below.

**Figure 3:**
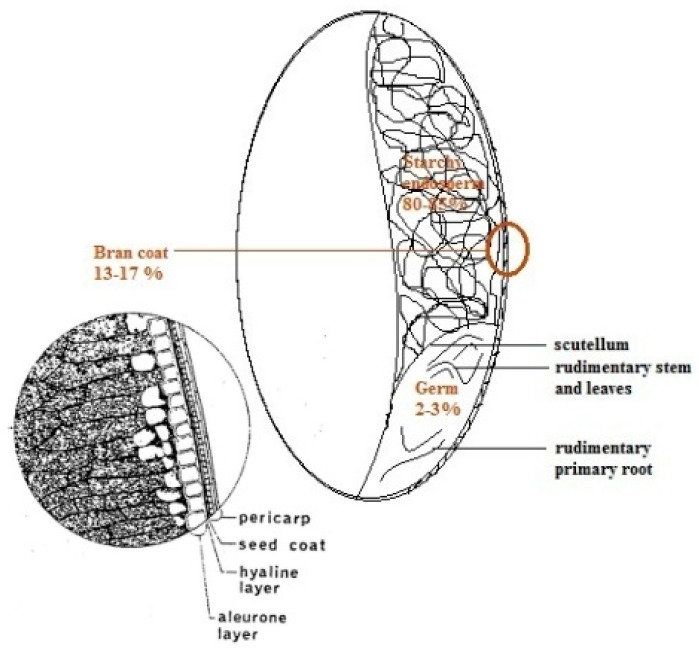
Schematic representation of wheat grain fractions (adapted from Reference [43]).

According to the literature, content of phenolic compounds is actually 15- to 18-fold higher in bran than that of the endosperm, which contains only 17% from the total phenolic content [42]. The bran coatings are as follows: The aleurone, the intermediate (hyaline) layer, the inner and outer pericarp [30], and they are illustrated in Figure 3 together with the other fractions of wheat grain. 

Bran is a key factor in determining whole grain health benefits, therefore the amount of total dietary fiber (TDF), insoluble dietary fiber (IDF), soluble dietary fiber (SDF), phenolic acids (FA: ferulic acid; PCA: *p*-coumaric acid; SA: sinapic acid; mg/kg) and total phenolic content (TPC) (mg gallic acid equivalents/kg) of the whole seed and the bran fraction of wheat, oat and barley were illustrated in Table 4.

According to Table 4, the amount of phenolic acids varies significantly both in whole grain and in bran; therefore the composition in food will be influenced by cereal variety and milling process [52]. The aleurone layer and the pericarp of wheat grain contain 98% of the total FA. Considering only the ferulic acid, for the consumption of 20 g wheat bran, an estimated intake of 100 mg total ferulate, 20 mg diferulates and minor amounts of other hydroxyphenolic acids will be achieved.

The amount of dietary fiber is higher in bran representing between 18.1% and 52.4% of the weight and the inverse correlation between its intake and chronic heart and gastrointestinal diseases has been constantly reported [19]. The physical structure of the cereal fraction also plays a major role in FA bioavailability and the health benefits found in animal and human intervention studies are likely related to FA bioaccessability and to the SDF/IDF ratio [19]. The contents of the principal phenolic acids (mg/g dry matter) of wheat fractions are illustrated in Table 5. 

Therefore, as shown in Table 5 and considering the data reported by Adom et al. [28], 83% of the total phenolic content is found in the bran/germ fraction of the whole meal flour, therefore the total phenolic content and, implicitly the total antioxidant activity, slowly decrease from the aleurone layer to the internal portions of the kernel [30]. The antioxidant activity of wheat grain fractions is inversely correlated to the aleurone content due to its high concentration in hydroxycinnamic acids, the major one being the ferulic acid. The bioavailability of the ferulic acid in grains is limited due to its strong boundary with indigestible cell wall material. A high variation in the phenolic acids and flavonoids was reported between wheat cultivars; therefore, a good approach for exerting a positive influence on public health would be the selection of wheat cultivars with higher levels of phenolics [46]. The phenolic acids and their relation to antioxidant activities may promote the development of cereal-based functional foods [53].

### 3.1. Biological Activities of Phenolic Acids in the Human Body

The bioaccessibility of whole grain phenolic acids strongly influence their biological activities [20,54] as their bound forms account for approximately 80% of the total content [49]. Whole grain phenolic acids may exhibit direct biological activities, such as an antioxidant, anticancer, anti-inflammatory, and antimicrobial potential [55].

#### 3.1.1. Antioxidant Effect

The most interesting property of cereal phenolic compounds is their antioxidant activity. According to the early study of Rice-Evans et al. (1996) [56], all phenolic acids have potentially antioxidant properties due to the presence of an aromatic phenolic ring. There are in vitro studies showing phenolic acids mechanisms of action: As free-radical scavengers, reducing agents, and quenchers of single oxygen formation [21,57]. Their antioxidant properties are explained by the electron donation and hydrogen atom transfer to free radicals [21]. In particular, hydroxybenzoic acids ability to activate a number of endogenous antioxidant pathways has led to an increased level of antioxidant enzymes, thereby decreasing oxidative stress and its implicit consequences, such as endothelial dysfunction and inflammation processes [58].

In general, the absorbed phenolic compounds are metabolized and conjugated [42]. For example, the ferulic and sinapic acids are absorbed as free and soluble forms present in the cereal [59]. The ferulic acid is esterified to arabinose segments in the cell wall. The release of bound forms of ferulic acid and other phenolics continues later in the colon through the fermentation process [60] and also through specific enzyme action, like gastrointestinal esterase which can release ferulic acid and diferulic acids from bran [61,62]. Phenolic acids from dietary fiber are converted in the colon to phenylpropionic, phenylacetic acid and benzoic acid (BA) metabolites [52,63]. Their certain antioxidant capacity and their absorption into the blood plasma has been reported [14,64]. Price et al. (2012) [64] studied the impact of a diet high in wheat aleurone on plasma antioxidant status and markers of inflammation by conducting a human intervention study for 4 weeks in 57 participants. Participants were healthy, older, overweight and consumed either aleurone-rich cereal products (27 g aleurone/day), or control products balanced for fiber and macronutrients as their habitual diets. The results indicated that, compared to the control, an intake of aleurone-rich products contributed with significant amounts of micronutrients and phytochemicals, which may function as antioxidants. Price et al. (2008) [65] analyzed phenolics and their antioxidant potential in human plasma and urine following the intake of a single meal of unprocessed wheat bran compared to a refined wheat cereal. Bran phenolics are relatively well absorbed and may contribute significantly to the antioxidant status. 

Nevertheless, the finding that the insoluble material can exhibit a remarkable antioxidant activity [66] offers future perspectives regarding the use of dietary fiber-phenolic compounds as health-related ingredients. Due to their survival in the gastrointestinal tract, bound phenolics may block the intestinal soluble radicals, which are responsible for many of the common pathologies [52].

In the study of Sevgi et al. (2015) [21], the antioxidant activity of individual phenolic acids was reported, with the ferulic acid showing the highest antioxidant activity compared to caffeic, chlorogenic, cinnamic, gallic, *p*-hydroxybenzoic, protocatechuic, rosmarinic, syringic, *p*-coumaric, and vanillic acids. A possible explanation for this could be the free radical scavenging potential of its three distinctive structural motifs [67]. In vivo, ferulic acid significantly blocked the free radicals, therefore preventing the oxidative stress correlated with alcohol and polyunsaturated fatty acids (PUFA)-induced toxicity [68]. Major antioxidant phenolic compounds in some common bran grains are shown in Table 6 below.

#### 3.1.2. Anti-Inflammatory Effect

The anti-inflammatory activity of phenolic compounds in humans is supported by a significant reduction of pro-inflammatory cytokines, whereas ferulic acid exhibits major effects on inflammatory messengers in several in vitro and in vivo models of inflammation [31].

In a recent in vitro study [70], the significant anti-inflammatory effect of the phenolic acids found in whole-meal flour of durum and wheat was reported. More precisely, this anti-inflammatory potential was exhibited in human colon cells (HT-29 human colon cells by measuring the levels of interleukin 8 (IL-8) and transforming growth factor β1 (TGF-β1)), resulting in an important intestinal health benefit. Another clinical study based on a randomized controlled group of obese subjects underlined the similar anti-inflammatory potential of phenolic acids from whole-wheat grain meals, precisely a reduction in plasma tumor necrosis factor-α (TNF-α) after 8 weeks and increased interleukin (IL)-10 only after 4 weeks with whole grain diet compared with refined grain diet. According to this study the whole-wheat based food had an increased content in fecal ferulic and dihydroferulic acid, which, is actually higher than in refined wheat-based products [71].

The wheat bran bioprocessing was associated with an increased anti-inflammatory capacity in an ex vivo lipopolysaccharide (LPS)-induced inflammatory response and this association implies a significant increase of phenolic acids bioavailability, as well as their metabolites, which possess immunomodulatory effects ex vivo [72].

#### 3.1.3. Antimicrobial Effect

Both classes of grain phenolic acids have antibacterial activity [73,74] and the recent literature presents the antimicrobial effects for hydroxycinnamates, such as caffeic acid, ferulic acid, and *p*-coumaric acid [20,75], whereas in ferulic acid most common metabolites are vanillin and 3-(4-hydroxyphenyl)-propionic acid. In the Taguri et al. (2006) study, 22 polyphenols were tested for their antimicrobial activity against 26 bacterial strains [76]. There is a structure-activity relationship between the strongest antibacterial activity (expressed as minimum inhibitory concentration (MIC)) for those polyphenols and a higher number of pyrogallol rings in their structure [73,76]. For example, the epigallocatechin-3-*O*-gallate (MIC 256 ± 141 μg/mL), castalagin (MIC 333 ± 157 μg/mL), epicatechin gallate (MIC 371 ± 252 μg/mL), pyrogallol (MIC 94 ± 91 μg/mL) had a strong antimicrobial activity against gram-negative bacteria, such as *Aeromonas hydrophila*, *Vibrio parahaemolyticus* and *Vibrio vulnificus*, due to the presence of pyrogallol groups. Therefore, the small mean MIC values (94–601 μg/mL) of the analyzed polyphenols, which contain pyrogallol groups, indicated the importance of this type of aromatic ring for a strong antibacterial activity. Phenolic acids have antimicrobial activity and show future perspectives for application as preservatives in food and food-packing materials as there were significant antimicrobial results when phenolic acids were incorporated into beef and food-packing materials [77,78].

## 4. Health Outcomes Associated with Consumption of Whole Grains and Bran Fractions

Epidemiological prospective, cohort studies and randomized controlled trials on humans show that the consumption of whole grain foods is protective against developing several diet and age-related diseases, such as type 2 diabetes, cardiovascular diseases, obesity, types of cancer [79,80,81,82], all of these diseases being associated with increased oxidative stress [83,84]. The various metabolic diseases are associated with lifestyle disorders, mainly a diet lacking in fiber and bioactive compounds (micronutrients and phytochemicals). It is generally agreed that the synergistic action of the compounds present in the bran and germ fraction of whole-grain cereals have a protective role [18,21,53] due to their antioxidant potential. The findings from the meta-analysis study of Aune et al. (2016) [85] sustain dietary guidelines that recommend increased intake (at least 3 servings/day or 75 g/day compared to less than 32 g or none in present) of whole grains to reduce the risk of chronic diseases.

### 4.1. Type-2 Diabetes (T2D)

According to the World Health Organization, the number of diabetic people increased from 108 million in 1980 to 422 million in 2014, whereas 1.5 million deaths were related to diabetes in 2014 [86]. Whole grain diets are correlated with a lower risk (20–30%) of developing T2D while the responsible compounds involve dietary fibers, vitamins, minerals and phenolic acids. Most phenolic acids diminish oxidative stress and inflammation, factors involved in the pathogenesis of T2D [87]. As the majority of phenolic acids are concentrated in the bran fractions, several human studies (men and women) reported an inverse correlation between the intake of wheat bran (bran-enriched products; cereal fibers) and the risk of type 2 diabetes [8,12,43,88] with a 20 to 40% lower risk [11,13,79].

A review carried out by The German Nutrition Society concluded that prospective observational studies consistently report that a high consumption of whole grain products or dietary fiber from cereal products contributes to a reduced risk of diabetes [89]. The recent systematic reviews and meta-analysis of prospective studies reported that whole-grain foods have a significant relationship with the risk of T2D, showing a consistent decrease of risk with increasing consumption [90,91]. A meta-analysis of six observational studies indicated a 26% lower risk of T2D in the population with an intake of 48–80 g whole grain/day (3–5 servings) compared to those with the lowest intakes [9]. The recent cohort study [92], reported a consistent association between high whole grain intake and lower risk of type 2 diabetes (an 11% and 7% lower risk per whole grain serving (16 g) per day) for all different cereals (wheat, rye, oats) and whole-grain products tested (rye bread, whole-grain bread, and oatmeal/muesli) for men and women. The recent meta-analysis of European prospective studies (European Prospective Investigation into Cancer and Nutrition-EPIC) done by the InterAct consortium [88] evaluated the association between intake of dietary fiber and type 2 diabetes, whereas their findings sustained the idea that the intake of total cereal fiber is inversely related to the risk of type 2 diabetes, suggesting that the association may be partially explained by body weight.

According to Malin et al. (2018), the mechanism pathway through which whole grains lower the diabetes risk involves a reduced post-prandial blood glucose and peripheral insulin resistance that are statistically linked to enhanced metabolic flexibility [93]. This mechanism is a property of soluble/viscous fiber. However, the major whole grain intake worldwide is based on whole grain wheat, which is low in this type of fiber.

Randomized controlled trials sustain also that the consumption of whole grains improves blood glucose control, may reduce fasting insulin levels, and reduces insulin resistance [9,36,79], indicating that a whole grain diet may increase insulin sensitivity, which controls blood sugar levels. The physical nature of the whole grain, as well as its level of processing, can affect the metabolic response to its intake. An early study suggested that the insulin responses increase as follows: Whole grains < cracked grains < coarse flour < fine flour [94], therefore, there is an inverse correlation between the forms of whole grain and the impact on glucose metabolism.

Recent observational data indicates that whole grains exhibit protective action against the development of type 2 diabetes, but long-term randomized controlled trials can elucidate the integral relationship between whole grains, dietary fiber, bioactive compounds and the metabolic response [95].

### 4.2. Obesity

In 2016, the World Health Organization reported that 1.9 billion people are overweight, whereas 650 million people are obese [96] meaning that compared to 1975, the obese population tripled. The intake of whole grains and whole grain based-products are inversely associated with the increased risk of obesity [9,97,98]. Miriam et al. (2012) [99] assessed the dietary phytochemical index over a 3-year change in weight, waist circumference, body adiposity index among adults, whereas their findings underlined the idea that a higher dietary phytochemical index could have favorable effects on the prevention of weight gain and reduction of body adiposity in adults.

In the early prospective cohort study, Liu et al. (2003) [100] conducted a research on more than 74,000 US women, over a 12-year period, to conclude if there is a correlation between whole grains and their dietary fiber intake and development of obesity. A 1.52 kg less was reported for women consuming consistently more whole grains and dietary fibers, while the research also indicated that obesity was directly correlated to the intake of refined grains [13].

Long-term prospective observational studies reported that a whole grain diet is associated with a lower risk of gaining weight over time [8,9,14,89], precisely three servings (48 g)/day may contribute to a lower body mass index (weight in relation to height), smaller waist circumference, and lower body fat levels.

When compared to control (non-whole grain foods), the evidence from randomized controlled trials suggest an inconsistency in the effects of whole-grain foods diet on body weight. For example, Pol et al. (2013) found no effect on body weight status of whole grain intake compared to refined grain intake. However, a beneficial effect of whole grain diet on the level of body fat was reported [101]. Larger and more long-term human intervention studies are required in order to identify if whole grain intake contributes solely to the healthier lifestyle status. 

There are several mechanisms mentioned by which consumption of whole grains may contribute to the weight management. Firstly, the fact that whole grain products possess a reduced energy density (kilocalories/unit weight). Secondly, their higher content in non-digestible carbohydrates compared to refined cereal foods [14,36], which increase satiety and therefore, the feelings of fullness [79]. 

In the review article of Sibakov et al. (2015) [43], a relevant human intervention study was reported for the whole-wheat grain, bran or aleurone intake and the reduced percentage of fat mass, namely the Kristensen et al. (2012) study [102] who showed a reduced percentage of fat mass in overweight or obese postmenopausal women.

Another meta-analyse comprising 50 trials on whole grain intake and body weight and adiposity correlation, reported healthful and statistically significant reductions in body mass index, waist circumference (a reduction of 2.7 cm), and central adiposity [103].

Due to the high rate of non-compliance within this topic, the most recent study by Kristensen et al. (2017) [104], implied an open-label researcher-blinded parallel design randomized trial. Within this trial, 179 overweight/obese women with a dietary low whole grain intake (<16 g/day) were randomized to a weight maintenance diet with refined-grain (RG) or whole-grain (WG) foods (80 g/day) for 12 weeks after an initial weight loss program over 8 weeks. Based on the results obtained, namely a very low rate of compliance, no conclusions about any effect of whole grain on weight maintenance was done, and the need to use objective measures of compliance in nutrition intervention studies was pronounced.

### 4.3. Cardiovascular Disease (CVD)

In 2015, the World Health Organization reported that 17.7 million people died from CVDs. The same organization estimated that by 2030, 23 million people will die of CVD annually around the world [105]. The most recent meta-analyses reviewing the prospective cohort studies on this topic reported that there is a consistent inverse association between dietary whole grain intake and the incidence of CVD [15,81,82,85,106].

Due to their bioactive content, the aleurone-rich products integrated into a routine diet may substantially reduce plasma concentrations of the inflammatory marker, C-reactive protein, which is an important risk factor for CVD [64]. One group of these bioactive compounds is represented by soluble fibers (most often consumed as β-glucan from oats). In addition to soluble fiber, whole grains are rich in phytochemicals, which compete with cholesterol for absorption in the small intestine, lowering LDL cholesterol, a triggering factor for CVD. Starting with the early meta-analysis of Brown et al. (1999) [107] which included 67 controlled trials, the oat fibers were reported to reduce total and low density lipoprotein (LDL) cholesterol. More precisely, 3 g soluble fiber from oats (3 servings of oatmeal, 28 g each) can decrease total and LDL cholesterol by approximately 0.13 mmol/L. Additionally, in the recent pilot study, the effects on health of 30 days intake of pasta enriched with 6% of β-glucan was investigated, resulting a significant decrease of LDL cholesterol with impact on CVD [108]. The intervention study carried out by Costabile et al. (2008) involved the administration of whole grain or wheat-bran breakfast cereals to healthy volunteers. The reported results indicated a more than two fold increase of FA concentration (from 2.2 mg/L at baseline to 5.70 mg/L) in the bloodstream after whole-grain breakfast cereal consumption. This increase may efficiently preserve in vivo some oxidation targets, such as LDL and triglycerides, thus leading to the prevention of CVD. In the randomized controlled trial of Tighe et al. (2010) [109], the whole grain consumption and CVD on middle aged people was examined. The results suggested that three portions of daily whole-grain foods intake reduced the risk of CVD by lowering blood pressure. There were three groups with a total of 233 participants, treated with refined, wheat, and oat plus wheat diets (one type of diet/group) for 16 weeks. At the end of this period, significant decreases of both systolic and diastolic blood pressure in the oat plus wheat group were observed. Compared to refined grains, whole grains, due to its high fiber content, were systematically reported as significantly reducing the risk of CVD by lowering the LDL and total cholesterol. For example, in the Holloender et al. (2015) [110] meta-analysis of randomized controlled trials the consumption of several whole grains, such as corn, rye, and brown rice, lowered risk of CVD by 20–25% while refined grains intake had no significant impact. Chen et al. (2016) [111] conducted a meta-analysis of prospective studies and concluded that there are significant inverse relationships between whole grain intake and mortality due to CVD. These findings encourage the recommendation of increasing whole grain intake to improve public health.

In the review article of Cho et al. (2013) [8], a systematic review based on a comprehensive PubMed search of human prospective studies (50 trials) including whole grain intake (as mixtures and food) with >25% bran and their risk association for type 2 diabetes, obesity and body weight measures and cardiovascular disease was conducted. The aim was to assess the contribution of bran in the reduced risk of T2D, obesity and CVD in human studies. Analyzed studies showed that the high intakes of mixtures of whole grains and bran had a higher positive impact in reducing the risk of CVD (a risk reduction of 7–52% for CVD mortality, CVD events, and heart failure) when compared to cereal fiber intake only (a risk reduction of 14–26% for CVD mortality and 22–43% for stroke). In Table 7 below, as main conclusions, the authors have summarized level of evidence among whole grains and bran intake and risk association of the above-mentioned diseases.

According to Table 7, there is reasonable evidence (moderate) for an inverse association of the intake of mixtures of whole grains and bran, as well as for cereal fiber intake and risk reductions for T2D and CVD, while the associations are inconclusive for only whole grains intake (limited). Whole grains are high in cereal fiber implying that they will prevent CVD, therefore a possible explanation for its limited evidence relates to the fact that data for whole grains alone are lacking in formation because of varying definitions among epidemiologic studies of what, and how much, was included in that food category. The problem may to some extent be methodological and whole grains may prevent diabetes and CVD, but cohort studies have not clearly shown this.

Campbell and Fleenor (2018) [112] conducted a human intervention study involving previously collected data from obese men (body mass index (BMI) ≥ 30.0 kg/m^2^) with cross-sectional baseline measures for habitual dietary intake and aortic stiffness (*n* = 22). The results suggested that whole grains intake, as a habitual diet, had a significant effect on reducing obesity-associated aortic stiffness, an important triggering factor for CVD. Responsible for this could be the synergistic effects of phytonutrients, micronutrients, and macronutrient in whole grains, as the total fiber content was not predictive of aortic stiffness (R^2^ = 0.06, *p* = 0.29). Fibers may have very significant health-related effects, but in this particular study, they did not account for the variation associated with the predictive power of whole grains in the determination of obesity-associated aortic stiffness.

### 4.4. Cancer

Considering the World Cancer Report by World Health Organization, cancer is perceived as the main factor contributing to morbidity and mortality, with an estimation of 14 million new cases and 8 million cancer-related deaths in 2012 [113]. The World Cancer Research Foundation report indicates that there is a strong evidence that consuming whole grains (90 g/day) decreases the risk of colorectal cancer, therefore, their recommendations suggest a diet rich in whole grains [27]. According to their report, the colorectal cancer prevention by whole grains consumption may be due to intestinal microbiota’s synthesis of short-chain fatty acids, reduced transit time or prevention of insulin resistance, as well as by antioxidant activity of phenolic acids which protect by binding carcinogens and regulating glycaemic response.

The consumption of whole grains and whole grain products has gained popularity because of their potential protection against cancer. This antioxidant effect is due to their phytochemicals that combat oxidative stress [114]. As mechanism pathways, whole grain phytochemicals regulate cellular signal transduction pathways and hence affect cancer cell behaviors such as proliferation, apoptosis, and invasion [115]. Bran antioxidants may concur to cellular protection while preventing oxidation damage at the cellular level. The potential of phenolic acids to inhibit cancer was attributed to the reduction in oxidative damage to cells and cell components [42,116,117].

In the early meta-analyses of Jacobs et al. (1998) [118], the case-control evidences supported the hypothesis that whole grain intake protects against various cancers. In the systematic review and dose-response meta-analysis of prospective studies conducted by Aune et al. (2011) [119], 25 prospective cohort and nested case-control studies of dietary fiber or whole grain intake and incidence of colorectal cancer were analyzed. The findings suggested that a high intake of cereal fiber and whole grains was associated with a reduced risk of colorectal cancer (0.83 to 0.97, I(2) = 0%). In the Larsson et al. (2005) [120] population-based cohort study of approximately 60,000 women, the risk association between whole grain intake and colorectal cancer was analyzed. The results indicated that high intake of whole grains (4.5 servings/day) may reduce the risk of colon cancer in women, when compared with a low intake (<1.5 servings/day). The results of a Scandinavian cohort study conducted by Kyro et al. (2013) [121] suggested the same findings, namely that whole grain products intake (and total whole grains) was associated with a lower incidence of colorectal cancer (incidence rate ratio [IRR], 0.94; 95% confidence interval [CI], 0.89, 0.99). Schatzkin et al. (2007) conducted a large prospective cohort study including 291,988 men and 197,623 women, where the dietary fiber and whole grain intake and the risk association of colorectal cancer was assessed. The results indicated a stronger association of high whole grains intake for rectal than for colon cancer.

Starting from the premise that evidence from previous reviews did not report risk cancer estimates for both whole grains and cereal fiber intake considering only case-control studies, Makarem et al. (2016) [122] conducted a systematic review of longitudinal studies on these criteria. The findings supported the idea that whole grains and cereal fiber may protect against gastrointestinal cancers, but confirmation in additional studies is required.

A meta-analysis of observational studies conducted by Lei et al. (2016) [123] investigated the potential of whole grain intake in the reduced risk of pancreatic cancer. They concluded that a high intake of whole grains might reduce the risk of pancreatic cancer. However, there is a need for more cohort studies and prospective studies for a stronger correlation.

Bound phenolic acids, due to their complex structure, may express their health effect by reaching the colon mostly undigested, where their unique antioxidant and anti-inflammatory activity is exerted, therefore contributing to a reduced risk for colorectal cancer [124,125].

## 5. Whole Grains and Bran Health Claims

Foods consisting of a minimum 6 g of wheat bran/100 g food can claim that they improve the fecal bulk. A food providing 10 g wheat bran/day, can claim it improves the transit time [126]. The first scientific opinion on whole grains and bran health claims was approved by European Food Safety Authority (EFSA) in 2010 [127], whereas the health claims involved the gut health and bowel function, weight management, blood glucose and insulin levels, blood cholesterol, satiety, glycemic index, digestive and cardiovascular health. Considering the worldwide different definition of whole-grain foods, it was concluded “… that the food constituent, whole grain, […] is not sufficiently characterized in relation to the claimed health effects …” Analyzing the data included in the report, the Panel decided “… that a cause and effect relationship cannot be established between the consumption of whole grain and the claimed effects considered in this opinion.” The US Food and Drug Administration has approved that certain foods containing at least 51% whole grains can claim to lower the risk of developing heart disease and certain cancers.

## 6. Whole Grains and Bran as Functional Foods

Recent findings on the health implications of whole grains, precisely their bioactive fractions, have underlined the potential status of wheat and other cereals as functional foods, which can reduce the risks for several chronic diseases [42,48,128]. Considering the annual consumption estimation for 2015 of cereals for 332 kg/person, their functional potential emerged across the research [129]. The majority of functional components of the wheat grains, namely fibers, vitamins, minerals, and phenolics, are mainly concentrated in the bran, therefore the use of bran in wheat-based products is considered an important approach to formulate functional wheat-based foods [18]. Recent research has highlighted that the functional potential of cereal-based food is related to their carbohydrate compounds, like β-glucan, arabinoxylan, inulin and to their bioactive compounds such as phenolics. Therefore, the Table 8 below summarizes the functional compounds together with their location in grain fractions.

The LDL oxidation model system for cardiovascular disease identification, as well as liposomes model system for cell membranes, can significantly contribute to the utilization of phenolic compounds in functional foods considering their health aim.

In particular, the functional food properties of whole grain bioactive compounds will be further discussed. In this respect, it is necessary to consider the following factors influencing their content, such as genetic variation, environmental factors and the form of compounds [49,140]. More precisely, it was underlined that the bound form comprises the highest content of the total phenolics [48]. However, genetic and genomic studies in cereal crops explain the path from genes to phenotypes. These valuable findings further allow for the cloning of genes responsible for biosynthetic pathways of functional food components together with comparative analysis and bioinformatics tools. New technological platforms are being increasingly used to investigate gene functions [42]. There were few experimental studies, which specifically reported an increase in phenolic acid content of cereal crops. Mao et al. (2007) [141] reported the highest content of ferulic acid (80.4%) from the total soluble phenolics by applying the gene “wheat oxalate oxidase” on maize lines. Considering the genetic and genome approach for whole-grain cereal functional food properties, more studies are required for exploring and establishing the phenolic acid synthesis pathway from a nutritional perspective. 

The incorporation of these compounds in food can be performed directly in its free form; however, micro- and nano-encapsulation techniques emerged as a very effective strategy to assure bioavailability of these compounds, helping to avoid the implications of food processing and ingestion [142,143].

## 7. Bioavailability

Regarding bioavailability from the nutritional side, it refers to the efficient use of nutrients and bioactive compounds by the body [31]. The biological effects or bio-efficacy of phenolic compounds is dependent on their bioavailability once consumed [144]. The low releasing rate of poorly-soluble bioactive compounds has resulted in low bioaccessibility and thus low bioavailability. Bioavailability of phenolic compounds is strongly influenced by the concentration and variation within the cell wall structure, glycosides forms in cells, molecular composition and the compounds-food matrix linkage [24].

The release of bioactives within the gastrointestinal fluids must be absorbed by the intestine epithelial cells before it is bioavailable for the organism, whereas the bioavailability rate of phenolic compounds is influenced by their molecular structure before absorption. During gastrointestinal digestion, chemical transformation, such as hydrolysis, reduction and oxidation may occur, therefore some bioactive compounds may undergo degradation under different pH or undergo isomerisation (cis-trans) before they become bioavailable [145].

### 7.1. Bioaccessibility and Intestinal Absorption

In the past, it was considered that due to these insoluble bounds there was a reduced polyphenolics bioaccessibility [54]. For instance, the free forms of ferulic and sinapic acid were greatly absorbed in the intestine from dietary high bran wheat, but their absorption may originate only from the free and soluble portions present in the cereals [18]. The recent studies present another metabolic pathway for wheat phenolics in the context of human digestion [146], namely that the intestinal microorganism are able to “eat” these strong bonds, thus contributing to an increased availability of phenolic acids. Therefore, it was admitted that the gut microbiota has a significant impact on polyphenol bioavailability and their bioactive potential [6,20,147], however, the variability among the individual gut microbiota composition, as well as the possible differences, which may occur in the daily polyphenol intake can influence their bioavailability and bio-efficacy. So far, there are only several microbial species identified as responsible for polyphenol metabolism, which, in fact, seem to be more personal than omnipresent [147]. However, the dietary polyphenols can also influence the composition of the gut microbiota population.

The cereal phenolic acids, namely the hydroxycinnamic acids, are bound to sugars, organic acids, and lipids through ester links. Due to a non-existence of esterases in the human tissues able to cleave these bounds, they are metabolized by colonic microbiota in the large intestine [47]. Even so, there is up to one third which is absorbed by the small intestine [148,149], whereas some hydroxycinnamic acids are polymers, therefore they present a high resistance to the action of LPH or CBG and cannot be absorbed in the small intestine. They reach the colon and its microbiota break the conjugating moieties resulting in aglycone forms [20]. Due to their metabolic process by the microbiota, different hydroxyphenylacetic acids result [150] and once a final derivative or aglycon has been up taken in the small intestine or colon, it is subjected to a following degree of phase II metabolism at enterocyte level, such as methylation, sulfation and glucuronidation. By the portal vein, they enter the blood level and undergo the next phase II metabolism, therefore, transformed into their conjugated form, they are taking the bloodstream pathway until they are excreted in the urine [151].

Regarding ferulic acid in particular, a human intervention study (six volunteers) conducted by Kern et al. (2003) [59] reported that FA was taken up in the plasma by ingesting a single meal of wheat bran cereals. Between 1–3 h after the intake, the FA concentrations reached the maximum level of 150–210 nM (mainly in the glucuronidated form), but it decreased slowly until 24 h. The possible explanations mentioned by the authors, sustain the hypothesis that, initially, only the soluble forms present in the high-bran breakfast cereals were mostly absorbed in the small intestine, where also their cleavage, release, and absorption took place. Further, both soluble and insoluble forms of hydroxycinnamic acids were released in the large intestine due to the action of bacterial enzymes being accessible to be further metabolized by the microflora or excreted via feces [146]. 

Nevertheless, the way the intestinal digestion influences the bioaccessibility of phenolic compounds is directed by the following factors: Intestinal enzymes and their action on the residual matrix, which can enhance the phenolic compounds; phenolic compounds degradation that can be accelerated by the oxygen and/or other transition-metal ions; absorption mechanisms [152]. In addition, the bioactive content and its bioaccessibility and bioavailability can suffer great modifications due to different processing techniques. 

### 7.2. Food Processing Influences the Bioaccessibility and Bioavailability

The role that food processing has on bioactive compounds is of major importance, as it comprises the first steps in the intensive journey toward targeted tissues [153]. On one hand, food processing can cause the reduction of phenolic compounds, and implicitly their amount in the final products, while on the other hand, processing can also determine chemical or physical modifications in food in such a way that enhances the release and absorption of phenolic compounds in gastrointestinal tract [32].

Several studies underlined the influence of processing and cooking on the bioavailability of total phenolic content and composition in whole grains. For example, the composition and the amount of phenolic acid can suffer significant changes when subjected to bakery and pasta-making processes, which might lead to major implications on the bioavailability of some compounds, like the ferulic acid [30].

Phytochemicals such as the phenolic acid found in the whole grains in free and bound forms change their structure during thermal and non-thermal processes. Nayak et al. (2015) [69] sustain that due to food processing high molecular weight phenolics break down into low molecular weight compounds, therefore enhancing the total phenolic content and antioxidant capacity. The findings of a previous study by Nagah and Seal (2005) [154] suggest that the cooking process did not affect the bound antioxidants within the food matrix. For example, in a recent study [23], the improved extrusions cooking treatment (IECT) over brown rice, wheat, and oat was investigated and the results suggested that total phenolic content and total antioxidant activity resulted from the free forms were decreased, while the total bound phenolic acids were significantly enhanced by 6.45%, 8.78%, and 9.10%.

Domestic cooking can enhance the phenolic acids extractability, as it is well known that their bioaccessibility is strongly influenced by polysaccharide interactions [155]. Roasting was observed to be the best domestic cooking method (amongst pressure cooking, boiling and microwave heating) to keep, and in some cases increase, the phenolic compounds bioaccessibility in pearl millet, finger millet, sorghum and wheat [156,157].

According to Hübner and Arendt (2013) [158] the malting process of cereal grains increases the amount of free phenolic acids. Alongside the release of bound compounds from the food matrix, the process of oxidation, polymerization or thermal degradation may occur to phenols. In the study of El-Sayed et al. (2013) [159], the baked products, when compared to the whole grain wheat flour, had an increased amount of free phenolic acids. A possible explanation could be the release of bound compounds during baking. However, in other studies, the baking process reduced the amount of free phenolic acids, being preceded by a fermentation step, which could explain the results [140]. When compared to bread products, it was concluded that free phenolic acids are in a higher amount and more stable in biscuits and muffins. This might be explained by the fact that phenolic acids are less exposed to oxidation in fat products than in other food systems [159]. To be completely objective, there are many other mechanisms [154], which may occur in parallel in the products during processing and may influence the content and composition of phenols. 

In the Li et al. (2007) study [160], the baking of purple wheat bran at 177 °C for 20 min did not influence the total phenolic content in the processed samples. Heat treatment (80 °C, 10 min) enhanced the total phenolic content and the antioxidant activity [161] by an increased bioaccessibility of the phenolic compounds [162]. Considering the oat groats experimental study of Bryngelsson et al. (2002) [163], steaming (100 °C, 1 h) and flaking (100 °C, 20 min) reduced caffeic acid, but increased ferulic acid and vanillin, whereas autoclaving (100–120 °C, 2.4 bar, 16 min) increased contents of vanillin, ferulic and *p*-coumaric acids. Drum-drying (8 bar steam pressure) of whole meal or rolled oats decreased all phenolics [163].

Extrusion cooking (120–200 °C) enhanced the phenolic content of oats [164] and sorghum bran [165], whereas wheat, barley and rye had an enhancement of 200 to 300% of vanillic, syringic and ferulic acids as free forms suggesting that hydrothermal processing may increase the releasing rate of phenolic acids and their derivatives from the cell walls [164]. This increase may be a consequence of the combination of high temperature, water-stress and wounding, however, the reduction of antioxidant activity and total phenolics of extrudates was reported for barley flour [166], in sorghum [167,168]. A possible explanation for the losses could be attributed to their low resistance to heat, evaporation and decomposition at these high temperatures [69]. Sultan et al. (2018) [169] reported the positive effect of gamma radiation (5 kGy) on total phenolic content of brown rice flour.

The cooking influence on fresh pasta obtained from durum wheat semolina enriched with common wheat debranned fractions was reported, whereas results showed a decrease of bound and free phenolic acids, no matter the flour matrices. By comparing the fresh with cooked pasta, a significant increase in bound phenolic acids was observed after the cooking process, a fact that can be explained by the release of bound ferulic acid due to the boiling water [170]. Another in vitro experimental study showed that the cooking process positively affected the antioxidant activity by the production of different compounds with antioxidant activity due to the Maillard reaction taking place during extrusion and drying processes of pasta [171]. In addition, the heat treatments have a deep impact on the chemical composition and physical properties of cereal dietary fibers. The mechanic rupture of the glycosidic bonds during the extrusion-cooking process could lead to an increase of soluble dietary fibers [29]. The increased antioxidant activity after processing could be due to the intrinsic properties of the food matrix, therefore, future research should concern the outer layers of grains due to the presence of bran, husk, testa, and aleuronic cells along with other processing parameters. 

### 7.3. Strategies to Increase Phenolic Content Bioavailability and Bioaccessibility in Whole Grains and Whole Grain Based-Products

Recent advances in food technology have made new processing methodologies available, which are able to add value to food products enhancing their nutritional and bioactive properties [32]. Due to the complexity of the bran matrix, which obstruct the responsible enzymes access to cleavage the boundaries, phenolic acids have very low bioavailability, namely a low release in the human gastrointestinal tract. Their increased accessibility has been demonstrated to be effective in enhancing their bioavailability [155].

Considering the following two conditions, food processing can be an intensely used tool for the enhancement of phenolic compounds bioavailability in whole-grain based foods: (1) A minimum degradation of phenolics through the process: (2) A minimum of matrix changes. Concerning the stability of phenolic compounds under food processing, the literature data show that in most cases phenolic compounds are sensitive to high temperature. Therefore, food processing without the use of high temperature acquires an advantageous position. Non-thermal processing [172] deserves separate mentioning, since it can be used as a substitute for the conventionally used thermal processing. When designing a strategy to increase phenolic compounds bioaccessibility and bioavailability, the choice of single or combined food processing technologies may be considered depending on the expected impact of each technology but, indeed, also on the requirements of the food product. The effects depended on the nature of the matrix and on the group of the phenolic compounds (total phenolics, total flavonoids or total phenolic acids). 

The enrichment with bran fractions, like aleurone that has a high bioactive potential and a strong antioxidant activity [173] is another important strategy. Experimental studies reported that an addition of 20% bran in the bread had a positive impact on its final sensory characteristics, like firmness, loaf volume, considering a bran fermentation of 16 h with yeast and lactic acid bacteria [174,175,176]. In order to obtain the best bran fractions, several pretreatments and new debranning processes have been developed [170,177,178], while the advantages of using these processes in the bakery and pasta industry are related to increased access of the bran fiber and phenolics, which deliver health-promoting effects, and to a reduced negative impact on the final taste. For instance, in the study of Blandino et al. (2013) [177] a 10% substitution with pearled wheat fraction improved, among others, the antioxidant activity and the total phenolic composition, keeping technological properties mostly unchanged. The use of intermediate pearled fraction in pasta and bread as a source of enrichment with phenolic acids was successfully reported [170,179].

#### 7.3.1. Bioprocessing Treatments

The bioprocessing treatments are an emerging way to improve the bioaccessibility of bioactive compounds and nutrients [51,155] as a feeding source for microorganisms [180]. Treatments like germination, fermentation, and enzymatic treatments have been recommended for enhancing the bioaccessibility, and therefore bioavailability of phenolic compounds of cereal foods [155,181]. Microbial fermentation is among the recommended fermentation treatments for the preparation of whole-grain based-foods rich in antioxidant phenolic compounds [182]. Lactic acid bacteria fermentation, or sourdough fermentation were successfully applied and can be used for preparation of functional cereal foods [183].

In a recent study, a different enhancement in the levels of nutrients, total phenolics, and antioxidant potential were reported after solid-state fermentation of wheat, rice, oat, corn, millet, broomcorn millet and sorghum by basidiomycete *Agaricus blazei Murrill* [184]. Another recent study, reported that sourdough-fermented bread, prepared from whole meal, was found to contain higher contents of free ferulic acid than those of yeast fermented bread. In addition, total antioxidant activity of sourdough-fermented bread was significantly higher than that of yeast-fermented bread [185].

The cell wall degrading enzymes method for the mobilization of bioactive molecules in the bran fraction represents a new pathway for grain enrichment and, in addition, enhances their level in cereal flour [181,186]. For example, ferulic content in bread supplemented with enzymatically treated bran was higher compared to untreated bran, therefore the enzymatically treatment increased the bioaccessibility of ferulic acids [187]. 

Nordlund et al. (2013) [188], applied successfully the following bioprocesses over rye and wheat bran: The cell-wall hydrolyzing enzymes (40 °C, 4 h) and the yeast fermentation (20 °C, 20 h) in order to change the bran structure. This bioprocess positively also affected the short-chain-fatty-acid formation and ferulic acid release in the colon model. Another study showed that the uptake of phenolic acids was 2-to-3-fold higher from bread with fermented bran, than from control bread with untreated bran [31]. Nordlund et al. (2012) [189] studied the phenolic acid metabolites resulted after in vitro gut fermentation of rye and wheat bran and aleurone fractions. The study implied the comparison between samples with and without an in vitro enzymatic treatment specific to the small intestine. The wheat aleurone samples yielded higher phenolic metabolites among which ferulic acid (phenylpropionic acid form) represented the majority. Anson et al. (2011) [72] approved the same findings, where the phenylpropionic acid was one of the main colonic metabolites of phenols. 

The enzymatic treatments, such as xylanase and feruloyl esterase, applied over a wheat aleurone fraction increased the amount of free ferulic acid by the cleavage of its conjugated form [190]. Furthermore, they found that the enzyme-treated aleurone fraction resulted in higher amounts of ferulic acid metabolites (e.g., phenylpropionic acid) after the addition of human fecal microbiota. Additionally, an increased absorption of ferulic acid in human health was reported due to the bioprocessing of rye bran when compared with native rye bran [191]. Yeast fermentation of germinated rye resulted in increased amounts of free phenolic acids compared with non-fermented rye germs [192]. Another recent example including the treatment with cellulase showed an increased total phenolics content and antioxidant of oat bran [193]. Xu et al. (2015) [194] reported an enhancement in physicochemical properties, total free/bound phenolics, and antioxidant potential of hulled and whole rice with the increased concentration of the added a-amylase up to 6% during extrusion. 

The genetic aspect of species and cultivars was also explored as a possible way for increasing the content of phenolic acid. Based on the existing studies, it was concluded that the genetic influence represents only a small factor as the environmental ones have the major decision on the final content of phenolic acids [48,195]. However, the use of an efficient breeding program was suggested for elite durum wheat as a possible solution for increasing the phenolic acid content in its germplasm [50].

An increase in the nutritive value, total phenolics content, dietary fiber content, and radical scavenging activity (DPPH) of the wheat flour with the increase in germination time of wheat grains was reported in several studies [196,197,198]. Therefore, soaking and germination have been recommended to enhance the nutritional value and potential functional effects of cereal grains and cereal-based products [199,200].

The solid-state fermentation has emerged as a potential technology for the development of bioactive products by increasing the phenolic content in cereal-based products. Therefore, these bioprocesses can become an important tool for the exploitation of cereal bran phenolic acids. 

#### 7.3.2. Mechanical Processing

The phenolic content, nevertheless all-bioactive content, was increased by the milling process modulation, which in fact decreases the particle size of whole-wheat bran and flour at the micronized scale [201]. Studies reported an addition of 5% micronized fractions in the fermentation process of wheat flour dough, in the bread-making process, for an improved and enhanced concentration of free amino acids, total phenols, dietary fiber, antioxidant activities of dough and improved sensory properties, while adding value in the functional food area [201,202]. 

A subsequent experimental study [203] reported two different methods for improving the bioavailability of phenolic acids in bran-rich breads, namely ultra-fine grinding and electrostatic separation. Within the study, in order to assess the bioaccessibility and bioavailability of phenolic acids, an in vitro gastrointestinal model assisted by a dynamic computer-control was employed. The results of this experimental study demonstrated the inverse correlation between the bran particle size and the bioaccessibility of phenolic compounds, namely the finer the particles, the more bioaccessible the phenolic acids. However, the results suggested also that only the free and conjugated forms were bioaccessible. When comparing the bioavailability of certain phenolic acids, higher quantity of SA was reported compared to that of the FA, mainly due to a better solubility of SA [203]. 

With respect to the electrostatic separation, this method implies a separation of each bran layer with its own composition, allowing, afterwards, for the use of only high phenolic contended bran fractions [204]. In this way, one can produce high phenolic content cereal foods. 

#### 7.3.3. Encapsulation

Encapsulation of phenolic compounds can sustain their stability during food processing and digestion, as well as enhance their absorption and extend their life in the bloodstream, resulting in improved bioavailability [187,205,206,207], therefore several studies have underlined nanocarriers as a good strategy to improve bioavailability of phenolics [208,209,210,211], such as lipid nanocarriers [212]. In 2017, Hu et al. [213] reviewed and analyzed in depth the encapsulation technology processes of phenolic compounds and their impact on bioavailability, where the main conclusions underlined the idea that nanocarriers may increase the bioavailability of phenolic compounds, mainly by enhancing their solubility, protecting from degradation in the intestinal tract, supporting the permeation rate in the small intestine, and, moreover increasing their contents in the bloodstream. Esfanjani et al. (2018) [214] concluded that nanocapsules based on lipid formulations provide a large surface area and have the potential to enhance solubility, improve bioavailability, and ameliorate controlled release of the nanoencapsulated phenolic compounds. For example, ferulic acid was entrapped into solid lipid nanoparticles (SLN) demonstrating a higher protective activity than free ferulic acid, against oxidative stress on human neuroblastoma cells (LAN 5) [215]. Another study presents the incorporation of ferulic acid within amaranth protein isolate: Pullulan ultrathin fibers using the electro-spinning process [216]. This encapsulation sustained a controlled release of ferulic acid and improved its antioxidant capacity during in vitro digestion (from 3.87 ± 0.04 to 15.16 ± 0.30 mmol trolox/g bioactive)). In the most recent experimental study of Granata et al. (2018) [217] hydroxycinnamic acids (HAs) were efficiently encapsulated in poly(ε-caprolactone)-based nanocapsules, then subjected to an in vitro digestion. Results suggested that these nanocapsules are able to carry out a controlled delivery of HAs resulting in potential increase of bioaccessibility in the intestine. The in vivo sustained release of ferulic acid encapsulated chitosan and its health effects on diabetes were reported by Panwar et al. (2018) [218], whereas results confirmed a four times enhanced bioavailability of encapsulated ferulic acid compared to the free form; a higher anti-diabetic potential of ferulic acid nanoparticles compared to the free form.

There is an emerging need for in vivo studies of the metabolism, absorption, and safety of phenolics loaded within nanocarriers in order to be suitable for using them in the formulation of functional foods. 

## 8. Future Perspectives and Outlooks

The majority of health-related compounds in whole grains are concentrated in the germ and bran that are discarded during production of white flour. Emerging evidence suggests that whole grain consumption has benefits beyond providing basic nutrition, a fact sustained by epidemiological studies, which suggest a protective action of a whole grain diet against cardiovascular disease, diabetes and cancer, as well in weight management. However, there are some inconsistencies in human intervention trials and mechanisms of action are still being explored and defined, but the findings indicate an inverse correlation between phenolic compounds (phenolic acids and flavonoid), fibers and health effects. In the future, the complex relationship between whole grains and health may be explained by well-designed intervention studies and could significantly contribute to the next generation of healthy cereal-based products.

European and Worldwide Health Authorities encourage the intake of whole-grain foods as part of a healthy diet, with disease prevention being a critical public health goal. 

Knowledge of the biochemistry, biological activity, bioavailability and genetics of whole grain phenolic acids is very vast, although more research is needed regarding the impact of grain phenolic acids on the gut microbiota and their mechanisms of action in humans.

By changing behaviors and perceptions from refined to whole-grain foods, an increased level of fiber, micronutrients and phenolic compounds would be reached, whereas health organizations and campaigns are incentive for whole grains consumption among consumers. The whole grain intake among populations can be facilitated by an increased variety of appealing whole grain products, informative labelling and sign posting of whole-grain foods. Thus, phenolic compounds are important natural bioactive compounds for preventing/inhibiting certain diseases.

General and standard definitions of ‘whole grain’ and ‘whole-grain foods’ is required, mainly considering its contribution in food manufacturers innovation, in consumer’s changing perception, in food-based dietary recommendations and public health policy.

The next generation of structured food for health is the key for an increased bioavailability of bioactive compounds and nutrients. Recent strategies investigated, like bioprocesses, encapsulation techniques, supporting the concept of health related functional whole-grain products due to an increased bioaccessibility of bioactive compounds and nutrients while keeping the sensorial attributes required by consumers. 

## Figures and Tables

**Table 1:** Nutritional profile of whole grains vs. refined grains (adapted from Reference [39]).

	Whole Wheat Flour	White Wheat Flour, 75% Extraction *	Whole Rye Flour	Rye Flour, 60% Extraction *	Whole Barley Grain	Pearl Barley
Carbohydrates, g (% of energy)	62 (75.6)	71 (80.6)	59.2 (71.4)	73 (85)	60.8 (72.8)	67 (79)
Protein, g (% of energy)	10 (12.2)	12.6 (14.3)	10 (13)	8 (9.3)	10.6 (12.7)	9 (10.6)
Fat, g (% of energy)	2 (5.5)	1.1 (2.8)	2 (5.8)	1 (2.6)	2.1 (5.7)	2 (5.3)
Dietary fiber, g	11	4	15	5	14.8	8.6
Vitamin B^1^, mg	0.4	0.07	0.4	0.15	0.31	0.03
Vitamin B^2^, mg	0.15	0.04	0.2	0.07	0.10	0.03
Vitamin B^3^, mg	5.7	1	1.7	1	5.2	3
Vitamin B^6^, mg	0.35	0.12	0.22	0.23	0.56	0.25
Vitamin B^9^, µg	37	22	78	28	50	20
Iron, mg	4	0.8	4	1.5	6.0	2
Zinc, mg	2.9	0.64	3	1.3	3.3	2
Magnesium, mg	124	20	92	51	91	44
Sodium, mg	5	2	5	10	4	5
B-glucan, g	0.7	0.08	1.9	n.d	4.4	4.0

* The percentage extraction of flour is the extraction rate, which is defined as the proportion of extracted flour with respect to the initial weight. The white flour is obtained for extraction rates equal or lower than 75% [40].

**Table 2:** Cinnamic acids derivates.

Cinnamic Acid Derivatives 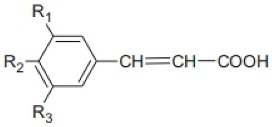	Substitutions		
	R1	R2	R3
Cinnamic acid	H	H	H
*p*-Coumaric acid	H	OH	H
Caffeic acid	OH	OH	H
Ferulic acid	CH_3_O	OH	H
Sinapic acid	CH_3_O	OH	CH_3_O

**Table 3:** Benzoic acids derivates.

Benzoic Acid Derivatives 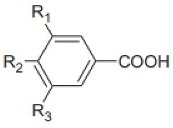	Substitutions		
	R1	R2	R3
Benzoic acid	H	H	H
*p*-Hydroxybenzoic acid	H	OH	H
Protocatechuic acid	H	OH	OH
Vanillic acid	CH_3_O	OH	H
Syringic acid	CH_3_O	OH	CH_3_O
Gallic acid	OH	OH	OH

**Table 4:** Bioactive compounds of wheat, oat and barley in whole grains and their bran fraction (g/100 g) (adapted by Vitaglione et al. (2008) [52]).

g/100 g	Wheat		Oat		Barley		Ref
	Whole	Bran	Whole	Bran	Whole	Bran	
TDF	11.6–17.0	36.5–52.4	11.5–37.7	18.1–25.2	14.6–27.1	-	[19,44]
IDF	10.2–14.7	35.0–48.4	8.6–33.9	14.5–20.2	12.0–22.1	-	[19,44]
SDF	1.4–2.3	1.5–4.0	2.9–3.8	3.6–5.0	2.6–5.0	-	[19,44]
FA	4.5–1270	942–5 400	359	-	168–723	2002–2017	[19,44]
PCA	0.2–37.2	100–457	-	-	4–374	2565–3367	[19,44]
SA	1.3–63	300	55	-	-	-	[19,44]
TPC	350–1505	2800–5643	1223	1950	1022–1193	-	[19,44]

TDF: Total dietary fiber; IDF: Insoluble dietary fiber; SDF: Soluble dietary fiber; FA: Ferulic acid; PCA: *p*-coumaric acid; SA: Sinapic acid.

**Table 5:** Major phenolic acids from wheat grain tissues (mg/g dry matter) (adapted from Reference [18]).

Wheat Fractions	FA	DHD	DHT	SA	*p*-CA	Total	Ref
Bran	5.26	1.01	0.24	0.25	0.09	6.85	[30,45]
Endosperm	0.10	0.03	0.00	0.01	0.00	0.14	[30,45]
Aleurone	8.17	1.07	0.11	0.44	0.21	10.00	[30,45]
Intermediate layer	5.92	0.91	0.07	0.08	0.07	7.05	[30,45]
Pericarp	8.18	5.12	1.21	0.01	0.04	14.56	[30,45]
Scutellum	3.48	0.37	0.03	0.01	0.01	3.90	[30,45]

FA: Ferulic acid; DHD: Dehydrodimers of ferulic acid; DHT: Dehydrotrimers of ferulic acid; *p*-CA: *p*-coumaric acid; SA: Sinapic acid.

**Table 6:** Phenolic antioxidants of cereal bran (adapted from Nayak et al. (2015) [69]).

Cereals Bran	Major Antioxidants
Wheat	Ferulic, vanillic, caffeic, coumaric and syringic acid
Barley	Protocatechuic acid, *p*-hydroxybenzoic acid, salicylic acid, vanillic acid, syringic acid, ferulic acid, coumaric acid, sinapic acid
Oat	*p*-hydroxybenzoic acid, vanillic acid,
Rye	Protocatechuic acid, *p*-hydroxybenzoic acid, vanillic acid, syringic acid, ferulic acid, *p*-coumaric acid, caffeic acid, sinapic acid

**Table 7:** Level of evidence between whole grains and bran intake and health benefits (adapted from Cho et al. (2013) [8]).

Source of Intake	Type 2 Diabetes (T2D)	Obesity	Cardiovascular Diseases (CVD)
Mixtures of whole grains and bran	Moderate	Moderate-Limited	Moderate
Cereal fiber	Moderate	Moderate-Limited	Moderate
Whole grains	Limited	Limited-Inadequate	Limited

Moderate = Evidence from multiple, well-designed, conducted, and controlled prospective cohort studies. Limited = Evidence from multiple prospective cohort studies from diverse populations, well-designed and conducted cross-sectional or case-controlled studies that have limitations. Inadequate = Evidence from studies that have one or more major methodologic flaws or insufficient data.

**Table 8:** Major functional food compounds in whole grains (adapted from Rawat et al. (2013) [48]).

Grain Fraction	Bioactive Compound	Whole Grain	Functional Potential	Reference
Pericarp, Testa and Aleurone	Arabinoxylans	Wheat, barley, rice, rye	Increase fecal biomass, enhance gut health and lipid metabolism	[130,131]
	Phenolic Acids and Flavonoids	All	Enhance redox potential; antioxidant and anticancer effects; hepatoprotective and antiaging properties	[132,133,134]
Aleurone	Minerals	All	Mg enhances cardiac health, sustains muscle properties; Fe, Zn and Cu sustain proper blood circulation, growth, development and body functions; Ca enhances bone health	[135,136,137]
	Inulin	Wheat, barley, rye	Prebiotic effect; enhances gut health and glycemic response	[138]
Endosperm	B-glucans	Oat, barley, rye	Decrease glycemic index; prebiotic effect	[139]
